# When in Doubt, Follow the Crowd? Responsiveness to Social Proof Nudges in the Absence of Clear Preferences

**DOI:** 10.3389/fpsyg.2020.01385

**Published:** 2020-06-18

**Authors:** Tina A. G. Venema, Floor M. Kroese, Jeroen S. Benjamins, Denise T. D. de Ridder

**Affiliations:** ^1^Department of Social, Health and Organisational Psychology, Utrecht University, Utrecht, Netherlands; ^2^Department of Psychology and Behavioural Sciences, Aarhus University, Aarhus, Denmark; ^3^Department of Experimental Psychology, Helmholtz Institute, Utrecht University, Utrecht, Netherlands

**Keywords:** nudge, social proof, uncertainty, conflict, preferences, indifference

## Abstract

Nudges have gained popularity as a behavioral change tool that aims to facilitate the selection of the sensible choice option by altering the way choice options are presented. Although nudges are designed to facilitate these choices without interfering with people’s prior preferences, both the relation between individuals’ prior preferences and nudge effectiveness, as well as the notion that nudges ‘facilitate’ decision-making have received little empirical scrutiny. Two studies examine the hypothesis that a social proof nudge is particularly effective when people have no clear prior preference, either because people are indifferent (in a color-categorization task; Study 1, *N* = 255) or because people experience a choice conflict (making shopping decisions about meat products; Study 2, *N* = 97). Both studies employed a social proof nudge to steer participants’ choices. The potential facilitating effect of the nudge was tested using a mouse-tracker paradigm that implicitly assessed experienced uncertainty during decision-making. Results showed that the nudge was effective in steering participants’ decisions; the facilitation effect (i.e., reduced uncertainty regarding the decision) was only observed for conflicting preferences, but not under indifference. A better understanding of when and how nudges can influence individuals’ behavior may help in deciding whether nudges are an appropriate policy tool for changing particular undesirable behavior.

## Introduction

With the realization that many societal issues such as climate change, obesity and personal debt are caused by a series of minor but imprudent individual decisions, governments have become increasingly interested in ‘nudges’ as a policy instrument to promote advantageous choices (Jones et al., 2013; Lourenco et al., 2016). Nudges are deliberate changes in the “choice architecture” (i.e., the way in which choices are presented) with the aim to facilitate the desirable choice without forbidding alternatives (Thaler and Sunstein, 2008). For example, to encourage sustainable behavior, a hotel might display a social proof message in the bathroom, stating that previous guests reused their towels (Goldstein et al., 2008). To promote healthier choices, governments may promote traffic labels on food packaging, to facilitate easy processing of percentages of sugar, fat and salt in relation to the Guideline Daily Amount (GDA) (Trudel et al., 2015). Although meta-analyses and systematic reviews consistently report that the majority of nudge interventions are effective, careful estimations indicate that the effect sizes are small (e.g., Skov et al., 2013; Broers et al., 2017; Szaszi et al., 2018; Hummel and Maedche, 2019).

Since the effectiveness of nudge interventions are usually assessed on a group-level, these small effect sizes may imply that an intervention is effective for some, but not all individuals (Olejnik and Algina, 2000). The few studies that have looked into moderators of nudge effectiveness suggest that people’s stance about the nudged behavior may impact whether or not individual choices are affected by the nudge (Szaszi et al., 2018; Hummel and Maedche, 2019). It has been shown, for example, that a default nudge to encourage towel reuse was less effective for people who were already concerned about the environment (Theotokis and Manganari, 2015). In a similar vein, the traffic light nudge to promote healthy food choices proved less effective for dieters than for non-dieters (Trudel et al., 2015). These findings suggest that people’s preferences for certain choices, stemming from their goals and values, might influence the effectiveness of nudges. Despite these initial findings, a systematic understanding of how antecedent preferences affect nudge effectiveness is still lacking. The present study aims to investigate the notion that nudges will be particularly effective in the *absence of a clear preference* for a particular choice option. Moreover, we test the hypothesis that in the absence of a clear preference nudges will facilitate the choice, i.e., makes the decision easier.

Despite the paucity in empirical research on the relation between preferences and nudges, the idea that nudges should not be effective when they do not speak to people’s preferences, is central to nudge theory that advocates “libertarian paternalism” (Thaler and Sunstein, 2008). Nudging is based on the idea that certain choices are better than others to improve well-being in the long-run, hence ‘paternalism,’ but only insofar people themselves are in agreement with the goals represented by these choices (see also Van de Veer, 1986). The ‘libertarian’ aspect requires that these choices are not enforced, but rather suggested. In fact, nudges are specifically designed for people who have adopted goals but fail to act upon them. To illustrate, a prompt that encourages people to take the stairs instead of the escalators should be effective for people who think they should be more active, but not for people with walking disabilities. The theoretical assumption that nudges should not be effective when they do not align with people’s preferences has only been tested in a few empirical studies. For example, one recent series of studies looked specifically at preferences that were either congruent or incongruent with the aim of the nudge intervention (Venema et al., 2019). A positioning nudge was employed that rearranged the presentation of small, medium and large cups of sugary beverages to encourage the selection of the smallest portion size. It was found that the nudge did not have additional effects for individuals with strong nudge-congruent preferences, such as a strong health goal (i.e., these individuals chose small portion sizes regardless of the presence of a nudge). Likewise, it was found that nudge-incongruent preferences were not hindered by the nudge; individuals who were thirsty chose larger portion sizes despite the nudge. Strong prior preferences, either in favor of or against the nudged option, thus rendered the nudge ineffective. A similar pattern was found in a study with an opt-out default nudge that automatically transferred people’s tax refunds into a savings account to encourage saving money. The nudge was not effective for people who already had plans to spend their refunds (Bronchetti et al., 2013; see Sunstein, 2017, for more examples). These initial studies demonstrate that nudge interventions are not likely to be effective when people have a clear preference that differs from the nudged alternative. An important follow-up to this work, then, is to examine whether and why nudges are particularly effective in the absence of a clear preference. The absence of a clear preference could either be due to being indifferent about the nudged choice or to experienced conflict as the different choice options appeal to different, contrasting, preferences. Indifference is characterized by a lack of perceived importance of the consequences of a decision. When people are indifferent they have no preference for a particular choice option, also referred to as *neutral* in the attitude literature (e.g., Kaplan, 1972). A good illustration of nudge effectiveness under indifference is provided in a study where the default printer settings were changed from single to double-sided printing (Egebark and Ekström, 2016). Although all participants had indicated that they knew that double sided printing is better for the environment, more than half of them did not have a clear preference for either single of double-sided printing. The change of the default setting resulted in a 15 percent reduction of paper use. This study illustrates the effectiveness of a nudge when people know of the consequences of their choice but are indifferent about the decision at hand.

One could say that conflicting preferences are the opposite of indifference regarding the meaning of the decision; while the decision is deemed unimportant when indifferent, the decision is highly important when experiencing conflicting preferences (e.g., Thompson et al., 1995; Yoo, 2010; Tang et al., 2014). However, both indifference and conflicting preferences are characterized by not knowing what to choose (i.e., uncertainty). An example of conflicting preferences might be spending time with family or spending time on a deadline at work; both options are deemed important but they preclude each other (Gollwitzer, 1993). Conflicting preferences can make a decision difficult and unpleasant, and resolving or reducing the conflict is therefore important (Van Harreveld et al., 2009). It has been suggested that environmental cues can tip the scale in favor of one of the preferences and thereby reduce the conflict (Tesser et al., 1983; Kruglanski et al., 2002; Griskevicius et al., 2006). This is well illustrated by a field experiment situated in a butcher shop, where customers were offered free samples of snacks while they were exposed to the smell of grilled chicken. While all customers were exposed to the smell that probably elicited a preference to take a snack, this setting only created a conflict for so-called restrained eaters who also have the incompatible preference to refrain from snacking. In the experimental condition a clearly visible poster was placed on the entrance door that advertised a recipe for a ‘slim dish’. It was found that customers who had a goal to restrain their eating ate significantly less when confronted with this environmental cue that reminded them of their goal, compared to the condition where there was no poster (Papies and Hamstra, 2010). This study illustrates that an environmental cue can be effective in resolving a choice conflict in favor of the sensible option.

To sum up, research has shown that nudge interventions are not likely to be effective when an individual has a strong preference in a choice setting. Elaborating on this finding, we posit that nudges are particularly effective in steering the decision when individuals have no clear preference. This preference framework offers a structure to understand the underlying working mechanism of nudges; a choice might be more difficult in the absence of a clear preference, either due to indifference or conflicting preferences. A nudge can make the decision easier by reducing choice uncertainty.

**FIGURE 1:**
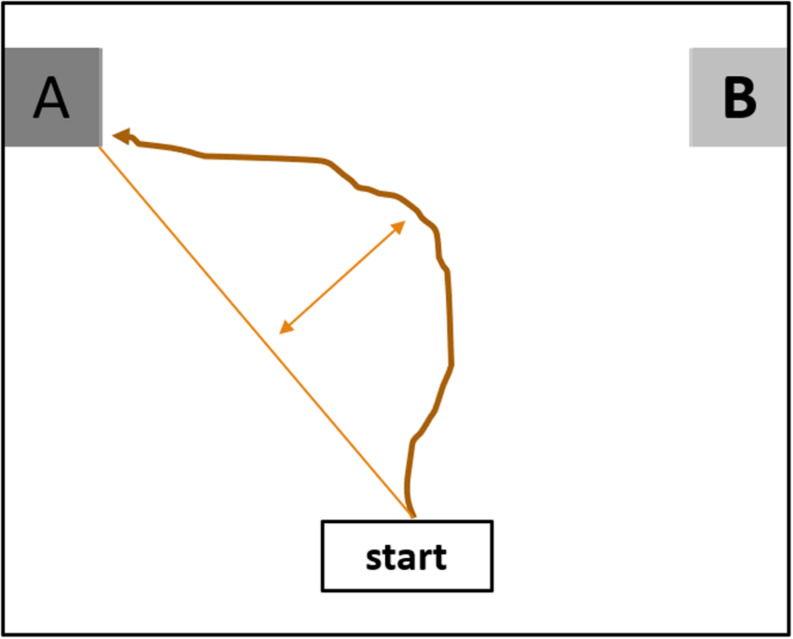
Mouse-tracker example trial.

To test the idea that a nudge facilitates decision-making by reducing uncertainty about the choice, the current studies will employ a mouse-tracker paradigm (Freeman and Ambady, 2010). This paradigm allows to measure implicit decision making processes as they unfold, without relying on self-report measures since those are generally biased by the outcome (Gillebaart et al., 2016). In this paradigm participants typically make a binary choice regarding an object by moving their mouse to one of the two choice options that are shown at the upper corners of the screen. The mouse-tracker calculates, amongst other measures, how much the participant strays from the most direct path from the object to their choice (maximum deviation). Specifically, maximum deviation is a summary of how much the *x-* and *y*-coordinates of the participant’s mouse movements differ from those of the most direct possible path, see Figure 1 for an illustration. Maximum deviation is often used as an objective approximation of how decisive the participant is in their choice, with a larger maximum deviation indicating more uncertainty (Schneider et al., 2015; Stillman et al., 2018). In the current study we test the facilitating effect of a nudge by measuring whether participants show less uncertainty (i.e., lower maximum deviation as measured with the mouse-tracker) when a nudge is present. Next to maximum deviation, the mouse-tracker measures how long it takes to make the decision (response time). As there are many factors that contribute to the duration of a response (e.g., reading speed, motivation to deliberate the decision, etc.), faster responses cannot unequivocally be interpreted as displaying more certainty when making the decision, nor can slower response times be straightforwardly interpreted as having *less* certainty while making a decision (Rubinstein, 2007; Konovalov and Krajbich, 2019). In the ambivalence literature, correlations between response times and maximum deviations are typically low (Schneider and Schwarz, 2017). However, we report response times next to maximum deviation for exploratory purposes. The current studies will be the first to employ mouse-tracker tasks to gain online insight into the underlying processes of decision-making in the presence of a nudge. Specifically, we predict that the presence of a nudge, compared to a control condition, has a facilitating effect on decision-making, as indicated by lower maximum deviation which we interpret as reduced uncertainty.

## Overview of Studies

Two experimental studies were conducted to investigate whether a nudge is effective in influencing a decision when participants have no clear prior preference. In study 1 participants categorized a range of colors into either green or blue. The effectiveness of the nudge was tested for the colors that are neither clearly blue nor green, hereby simulating a situation in which people experience no clear preference when the consequences of the decision are relatively unimportant and thus mimicking indifference. In study 2 the effect of a nudge under conflicting preferences was investigated: (non-vegetarian) participants had to accept or reject meat products in a grocery shopping task. The consumption of meat is known to invoke conflicting preferences because people might simultaneously endorse reasons to choose meat (because they like the taste) as well as reject meat (because they know that meat consumption is harmful for animals and the environment) (Loughnan et al., 2010). The magnitude of this conflict is stronger for some than others. The effectiveness of the nudge was tested for participants who vary in the extent to which they experience conflicting preferences, where those who are highly conflicted were expected to be more uncertain about their choice. Both studies employed a social proof nudge (e.g., Goldstein et al., 2008; Salmon et al., 2014) to steer participants’ choices. This nudge simulates or highlights the descriptive norm regarding a decision by purposefully showing what other people have chosen (Stok et al., 2014). In the social proof conditions participants were told that the responses of previous participants would be shown in a bar graph during the task. Our first hypothesis is that the nudge will be effective in steering the choice when people have no clear prior preference. Secondly, we hypothesize that in the case that there is no clear preference a nudge facilitates the decision, as indicated by reduced uncertainty (i.e., lower maximum deviation scores on the mouse tracker task).

## Study 1

Study 1 simulated a choice situation in which people have no clear preference because they are indifferent about the choice options. Participants were asked to judge whether a presented color square is either blue or green. In some of the trials the decision for blue or green was easy because participants can be certain about the correct answer (i.e., the color is clearly blue or green). In the trials of interest, however, participants were expected to be uncertain about their decision because the color was ambiguous. In these trials, the effectiveness of the social proof nudge will be tested. It was expected that the social proof nudge will guide participants’ decisions such that when it is suggested that previous participants mostly chose ‘green’ (or ‘blue’) participants will also be more likely to choose for green (or blue) compared to when no nudge is present. The hypothesized facilitation effect is investigated by comparing the differences in mouse-tracker trajectories between conditions, where we expected that in the nudge condition participants would display smaller maximum deviations as compared to the control (no-nudge) condition. For exploratory reasons, state self-control and participants’ general tendency to doubt their decisions were assessed, as these factors might influence the effectiveness of the nudge. In addition, participants’ identification with the social proof reference group was measured.

### Method

#### Participants

Two hundred seventy-seven Mturk workers with an approval rate higher than 97% participated in this study and were compensated with 0.37 dollar cents. Data from 20 participants whose mouse-tracker data could not be matched on completion time and IP-addresses to the questionnaire data and the data from two participants who indicated to be color-blind were excluded from analysis. Two hundred and fifty-five participants (44.7% female) were included in the analysis, with 84 participants in the control condition, 86 in the social proof blue and 85 in the social proof green condition. The majority of the participants were right-handed (93.4%) and the average age was 36.31 years (*SD* = 11.50).

#### Design and Procedure

The present study used a 2 (trial type: critical and non-critical) × 3 (condition: social proof blue vs. social proof green vs. control) mixed design, with the former as a within-subject factor and the latter as a between-subject factor. The study was approved by the Ethics Review Board of the Faculty of Social and Behavioural Sciences. Participants were randomly assigned to one of the three conditions. After providing consent participants filled out the State Self-control Scale. Then they continued with the mouse-tracker task, followed by the General Doubt Questionnaire, and a funneled debriefing that included manipulation checks of the nudge. Participants were asked for their age, gender, color-blindness and handedness. Upon finishing participants were thanked and prompted to leave any comments or questions.

### Materials

#### State Self-Control Scale (SSCS)

The SSCS was assessed as an approximation of the available cognitive resources, since individuals low in state self-control might be particularly prone to be responsive to nudges (e.g., Brownstein, 2003; Salmon et al., 2015). The state self-control scale consists of 10 items, e.g., “I would want to quit any difficult task that I was given” (Twenge et al., 2004). Participants responded on a 7-point Likert scale ranging from 1 (*not true*) to 7 (*very true*). The items 5 and 7 were reverse coded, the average was calculated to provide a state self-control score. A lower score indicates higher state self-control. Cronbach’s α was 0.88.

#### Mouse-Tracker Task

The mouse-tracker was programmed in JavaScript and implemented on the LimeSurvey platform (version 2.05 + build 141229). Participants categorized 33 color stimuli from the Farnsworth- Munsell 100-Hue test (e.g., Cranwell et al., 2015) as either “green” or “blue” by moving their mouse to the choice-box (“green” corresponding to the left box, “blue” corresponding to the right box). The non-critical trials consisted of 26 stimuli (12 green and 14 blue). Based on a pilot study, 7 critical stimuli were selected for which participants had no clear preferences (these could be described as “ocean/aqua” or “turquoise”)^1^. The dependent variable Choice Likelihood was constructed by coding the choice for green as 1 and the choice for blue as 0 for the critical trials and averaging the scores. A score of 0.71, for example, corresponds to choosing 5 out of 7 times for green in the critical trials. All stimuli were presented in a random order. To assess the extent of uncertainty an average maximum deviation was calculated for all trials. Response times are reported for exploratory purposes^2^.

#### Nudge

**FIGURE 2:**
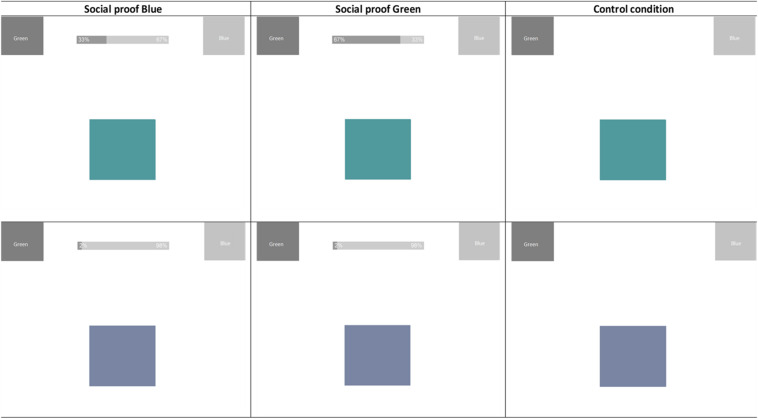
Example trials critical stimuli (top half) and non-critical stimuli (bottom half) Study 1.

The social proof nudge was designed as a bar graph at the top of the screen that represented the choices of alleged previous participants (Figure 2). Participants in the social proof conditions were explicitly told that the bar represented a summary of the answers of previous participants. The two social proof nudge conditions (green and blue) only differed from each other in the critical trials. For the critical trials the bar graph showed “previous ratings” between 62 and 82% in favor of either green, in the social proof green condition or blue, in the social proof blue condition. Both social proof conditions had the same bar graphs for the non-critical trials, with the ratings ranging from 85 to 99% in favor of the unambiguous color (i.e., in favor of green for the green trials and in favor of blue for the blue trials, see bottom half Figure 2). The control condition had no bar graph at the top of the screen.

#### General Doubt Questionnaire (GDQ)

The GDQ measured the daily doubt experience of participants. This measure was assessed for exploratory purposes. It consisted of eight statements about doubt, which participants evaluated to the extent it applied to them, (e.g., “I find it difficult to make decisions,” “I can choose well when faced with multiple decisions” [rev. coded]). Participants responded on a 5-point Likert scale ranging from 1 (*false*) to 5 (*true*). The items 3, 5, and 7 were reverse coded. The GDQ had a Cronbach’s α of 0.90. A higher score indicates that the participant generally doubts more when making decisions.

#### Funneled Debriefing

First, all participants were asked whether they had seen the answers of previous participants during the choice task, and if so, found them useful, answered by yes or no. Next, identification with other Mturk workers was assessed with two items. Participants indicated to what extent they saw themselves as a member of the Mturk Workers community and to what extent they identified with Mturk workers on a visual analog scale ranging from 0 (*not at all*) to a 100 (*very much*). The two items were highly correlated, *r* = 0.78, *p* < 0.001. An identification variable was calculated by the average of the two items.

### Results

#### Randomization Check

Separate one-way ANOVA’s showed that participants across the three conditions did not differ in age, state self-control, identification with other Mturk workers or on the general doubt questionnaire, all *p’s* > 0.141. A Chi-square test showed that gender and handedness did not differ per condition, *p*’s > 0.172, indicating successful randomization.

#### Manipulation Check

To check if the critical trials invoked more uncertainty than the non-critical trials, maximum deviation scores were compared in the control (no nudge) condition. A paired *t*-test indicated that participants exhibited more uncertainty, as measured with maximum deviation, toward the critical stimuli (*M* = 144.44, *SD* = 79.85) than the non-critical stimuli, *M* = 120.83, *SD* = 63.02, *t*(83) = 4.16, *p* < 0.001. The findings on the maximum deviation were corroborated by those for response time. Participants took on average 1561.08 ms (*SD* = 534.26) for the non-critical trials to make a decision, whereas they took significantly longer during the critical trials, *M* = 1867.15, *SD* = 749.83, *t*(74) = −5.45, *p* < 0.001. The successful manipulation of uncertainty had a medium effect size for maximum deviation, *d* = 0.46 and a large effect size for response time, *d* = 0.63.

As for the nudge manipulation, approximately half of the participants in the social proof conditions indicated to have seen the social proof bar, 51.1% in the social proof blue condition and 51.8% in the social proof green condition. Surprisingly, from those who had indicated to have seen the social proof nudge, participants in the social proof green condition (62.8%) found it significantly more helpful than the participants in the social proof blue condition (37.8%), χ^2^(1) = 5.50, *p* = 0.019. The participants who had seen the social proof did not differ between the experimental conditions on identification with Mturk workers, *p* = 0.369. Given the large proportion of participants who had not noticed the social proof nudge, it was decided to run the main analyses twice, once with all the participants and once without the participants who failed to notice the social proof nudge.

#### Main Analysis

##### Effect of social proof nudge on choice

**FIGURE 3:**
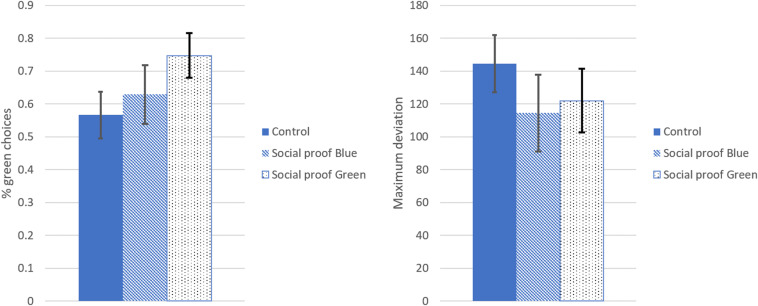
Results Study 1.

It was hypothesized that in the absence of a clear preference the nudge would influence the outcome. First, a one-way ANOVA was run with Choice Likelihood as the dependent variable and condition as predictor variable. When all participants were included, regardless of having seen the nudge, the effect of condition was marginally significant, *F*(2,254) = 2.43, *p* = 0.090, $\eta_{\text{p}}^{2}$ = 0.02. Excluding the participants who had not noticed the nudge led to a significant effect of condition on the choice, *F*(2,169) = 5.36, *p* = 0.006, $\eta_{\text{p}}^{2}$ = 0.06. Pairwise comparisons with LSD correction showed that participants in the social proof green condition (*M* = 0.74, *SD* = 0.22) were significantly more likely to choose green than participants in the control condition, *M* = 0.57, *SD* = 0.33, *p* = 0.001, Cohen’s *d* = 0.65. Participants in the social proof blue condition (*M* = 0.63, *SD* = 0.30) did not differ significantly from the control condition, *p* = 0.255, Cohen’s *d* = 0.20 (see left pane of Figure 3). The nudge was effective in steering the choice to green, but not to blue.

##### Facilitation by the nudge

It was hypothesized that the social proof nudge would yield a facilitation effect, indicated by a lower maximum deviation (i.e., lower uncertainty) in the critical trials in the nudge conditions compared to the control condition. First, when including all participants, a one-way ANOVA with maximum deviation in the critical trials as outcome variable and condition as predictor indicated no significant difference between the conditions, *F*(2,255) = 0.90, *p* = 0.408, $\eta_{\text{p}}^{2}$ = 0.01. Excluding the participants who had not noticed the nudge led to a marginally significant effect of condition on maximum deviation, *F*(2,169) = 2.71, *p* = 0.069, $\eta_{\text{p}}^{2}$ = 0.03. Participants in the control condition displayed larger maximum deviations (i.e., larger uncertainty) (*M* = 144.44, *SD* = 79.85) than the participants in the social proof blue condition (*M* = 114.51, *SD* = 77.80) and the social proof green condition (*M* = 121.99, *SD* = 63.00). Although the hypothesized trend was observed, the difference did not reach statistical significance (see right pane in Figure 3).

For exploratory purposes, a MANOVA was run with response time and maximum deviation in the critical trials as dependent variables. First, when including all participants this analysis showed a significant difference for response time between the conditions, *F*(2,239) = 4.00, *p* = 0.020, $\eta_{\text{p}}^{2}$ = 0.03. A LSD corrected *post hoc* test showed that participants in the social proof blue condition (*M* = 2326.50, *SD* = 1119.17) took significantly longer to make a decision in the critical trials than the participants in the control condition (*M* = 1903.01, *SD* = 784.38), *p* = 0.005. The social proof green condition (*M* = 2138.07, *SD* = 883.92) did not differ significantly from the control condition (*p* = 0.140), nor from the social proof blue condition, *p* = 0.179. However, the effect of response time disappeared when the participants who failed to notice the nudge were excluded, *F*(2,157) = 1.46, *p* = 0.235, $\eta_{\text{p}}^{2}$ = 0.02. The nudge does not seem to influence the uncertainty that the stimuli invoked under indifference.

#### Exploratory Analysis Helpfulness

To understand why some participants found the nudge helpful, a one-way ANOVA was run with maximum deviation for the critical trials as outcome variable and helpfulness (yes vs. no) as predictor. Helpfulness was significantly related to the amount of uncertainty that was experienced in the critical trials, *F*(1,90) = 10.51, *p* = 0.002, $\eta_{\text{p}}^{2}$ = 0.11. Participants who had considered the nudge helpful in hindsight, had experienced more uncertainty in the critical trials (*M* = 143.58, *SD* = 73.38) than the participants who did not find it helpful, *M* = 97.07, *SD* = 62.64. The same analysis with response time as dependent variable indicated that response time was not related to finding the nudge helpful, *p* = 0.753. So even though the nudge did not influence uncertainty, uncertainty did seem to influence the perceived helpfulness of the nudge.

### Discussion

The aim of study 1 was to test the effectiveness of a nudge when people are indifferent about choice options and specifically, whether a nudge makes uncertain decisions easier. It was demonstrated that the social proof nudge influenced participants’ choices in the critical trials in the direction of green, but not toward blue. One possible explanation for this discrepant finding could be that, despite a pilot study, the critical stimuli on average were more optically green than blue, resulting in participants having a slight response tendency for green. This idea is corroborated by the choices from participants in the control condition who were also more likely to choose green over blue and by the finding that participants in the social proof blue condition rated the nudge as less helpful compared to participants in the social proof green condition. A similar tendency toward green was also found in previous color categorization research with a mouse-tracker (e.g., Huette and McMurray, 2010), also ruling out the location of the answering category as explanatory factor (i.e., left ‘green’ and right ‘blue’). Taking this methodological issue into account, we conclude that the first hypothesis, that a nudge is effective in the absence of a clear preference, was partially supported. The second hypothesis that a nudge facilitates a decision by reducing uncertainty is not supported under indifference. While study 1 pertained to choices that had little personal relevance, study 2 tests the effectiveness and facilitation effect of a nudge under conflicting preferences where personal relevance is high.

## Study 2

In study 2 we make use of the *meat-paradox* phenomenon (Loughnan et al., 2010) to invoke conflicting preferences in a choice situation. The consumption of meat has been well documented in the literature as causing ambivalent feelings (e.g., Berndsen and Van der Pligt, 2004; Buttlar and Walther, 2018). On the one hand, people like the taste of meat and on the other hand they experience discomfort when thinking about what happened to the animals or the consequences for the environment. Because the magnitude of this conflict differs per individual, a within-subjects design was used to investigate the effectiveness of the nudge in steering the choice toward refraining from meat consumption, in line with the nudge-for-good philosophy that advocates nudging toward decisions that benefit both the individual as well as society (e.g., Bovens, 2009; Sunstein, 2015).

### Method

#### Participants

An *a priori* power analysis using G^∗^Power 3.1 based on an expected correlation between the blocks of 0.80, indicated a minimal sample size of 84 to achieve statistical power of 0.80 to detect a small effect size (*d*) of 0.10 for a within-subjects design (Faul et al., 2007). 120 participants were recruited via leaflets and posters on the university campus and through social media for a study advertised as online grocery shopping. 20 participants were excluded from the analyses because they adhered to a vegetarian or vegan diet, and three participants were excluded from analyses because due to a technical error no mouse-tracker data was collected. Leaving a final sample size of 97 participants (76.3% women; mean age 22.15 years, *SD* = 4.32). Participants received either partial course credit or two euros in exchange for their participation.

#### Procedure and Design

This study had a within-subjects design (control vs. social proof nudge) with percentage of chosen meat products as the dependent variable. Participants were told that the aim of the study was to select supermarket products for an alleged future experiment, and that they would be presented with 100 new products and 100 previously tested products that needed validation. After providing informed consent participants filled out a questionnaire that assessed demographics (gender, age, height, weight, diet type, and education level). Frequency of meat consumption was assessed to corroborate the diet type. Consecutively, self-reported ambivalence and attitude toward meat were assessed. To conceal the true aim of the study, the self-reported ambivalence and attitude questions were also included for snacks and non-organic fruit/vegetables. Familiarity with the supermarket and current hunger and thirst were assessed before participants proceeded to the shopping task. This shopping task was an adaptation of the mouse-tracker task in study 1. In study 2 participants indicated in the mouse-tracker program for each of a 100 “new” products whether they would ‘select’ or ‘reject’ that product. This first block of “new” products served as the control condition. They then proceeded to the second block in which they again indicated ‘select’ or ‘reject’ for 100 products that were allegedly tested before. The ratings of these alleged previous participants were shown in the top half of the screen as a percentage bar to serve as a social proof. Before starting the second block participants were explicitly told by the experiment leader that the bar represented a summary of the answers of previous participants. After the second block participants were probed for the conjecture of the study and received a funneled debriefing that served as a manipulation check. The study was approved by the Ethics Review Board of the Faculty of Social and Behavioural Sciences.

### Measures

#### Self-Reported Ambivalence

The extent to which respondents’ feelings toward eating meat were conflicted were measured with three items on an 11-point scale ranging from 0 (*feel no conflict at all, feel no indecision at all, and completely one-sided reactions*) to 10 (*feel maximum conflict, feel maximum indecision, and completely mixed reactions*) (Priester and Petty, 1996; Berndsen and Van der Pligt, 2004). Cronbach’s alpha was 0.82. The average self-reported ambivalence score was *M* = 4.14, *SD* = 2.31.

#### Attitudes Toward Meat

Attitudes were assessed to corroborate the self-reported ambivalence. Previous studies have shown that conflicting feelings about meat correlate highly with negative attitudes (Berndsen and Van der Pligt, 2004). Five semantic differential scales were used as measurement ranging from 0 to 100 and had the labels bad–good, unpleasant–pleasant, against–in favor of, unfavorable–favorable, and negative–positive (Berndsen and Van der Pligt, 2004). Cronbach’s alpha was 0.90. The variable attitude was constructed by calculating the mean of the five items. A higher score indicates a more positive attitude toward eating meat. The average attitude score was *M* = 54.09, *SD* = 19.57.

### Materials

#### Mouse-Tracker Task

**FIGURE 4:**
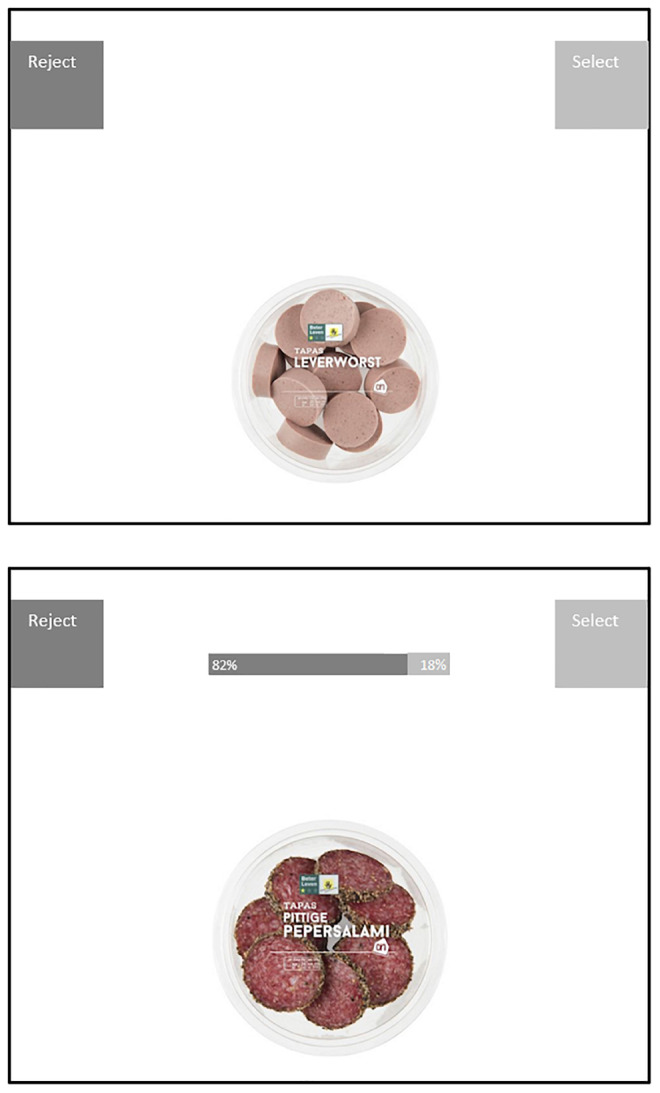
Example of trials Study 2.

In each block participants decided for one hundred products (40% meat and 60% non-meat filler trials) whether they would reject or select that product by moving the mouse to one of these two options. In each trial, an image of the product was shown that revealed no nutritional or price information. The products in the control and social proof condition were matched such that they were highly similar but still different, for example, lasagne Bolognese in the control condition and lasagne with minced beef in the social proof condition, see Figure 4 for example trials. Within each block all product trials were presented in a random order.

#### The Nudge

In the social proof condition all critical trials (the meat products) were accompanied by a social proof bar that displayed 82% reject versus 18% select. For the purpose of a coherent cover story, products in the non-critical trials (the non-meat products) were also presented with a social proof bar displaying a distribution of select-reject ratio’s, such that participants should be encouraged to reject roughly half of the products (i.e., respectively, reject- select 10–90; 32–68; 54–46; and 66–34%). Non-critical trials were not analyzed; the dependent variable was the percentage of chosen meat products per block^3^.

### Results

#### Main Analysis

##### Effect of social proof nudge on meat choice

**FIGURE 5:**
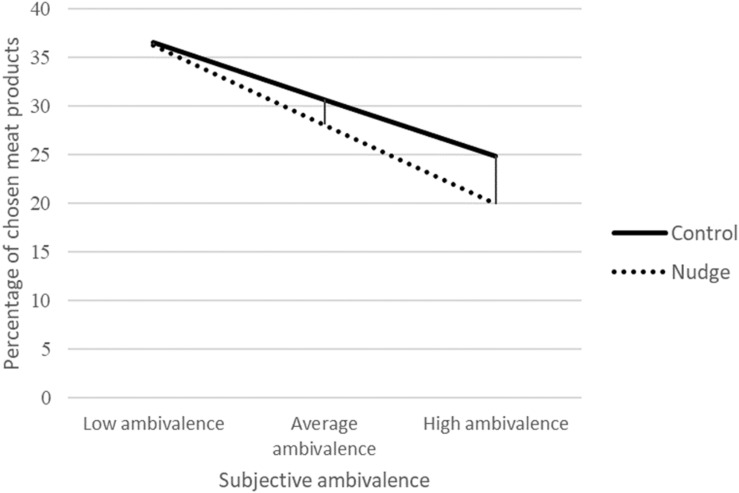
Results Study 2 on choice.

To test the first hypothesis that a nudge is effective in steering a decision when people have conflicting preferences, a repeated measures ANOVA was performed comparing the percentage of chosen meat products in the control condition to the nudge condition. Self-reported ambivalence was added as a covariate. There was no significant main effect of condition, *F*(1,95) = 0.97, *p* = 0.326, $\eta_{\text{p}}^{2}$ = 0.01; in the control condition 30.72% (*SD* = 17.72) of the meat products was selected and in the nudge condition participants chose 28.11% (*SD* = 19.71) of the meat products. This indicates that the social proof nudge had no effect on the selection of meat products on a group level. However, there was a significant main effect of self-reported ambivalence, *F*(1,95) = 16.30, *p* < 0.001, $\eta_{\text{p}}^{2}$ = 0.15. Participants who experienced more self-reported ambivalence were less likely to select meat products overall, *R* = −0.383, *p* < 0.001. Moreover, the Condition × Self-reported ambivalence interaction was significant, participants who experienced higher ambivalence concerning meat consumption were more affected by the social proof nudge that suggested to reject meat products, resulting in fewer meat choices, *F*(1,95) = 8.51, *p* = 0.004, $\eta_{\text{p}}^{2}$ = 0.08. These findings are in support of the hypothesis that a nudge is particularly influential in steering decisions when people have no clear preference, now demonstrated by conflicting preferences. Figure 5 shows the average choice for the meat products in percentages across the conditions for −1 *SD*, the average and +1 *SD* of subjective ambivalence.

##### Facilitation effect of the nudge

**FIGURE 6:**
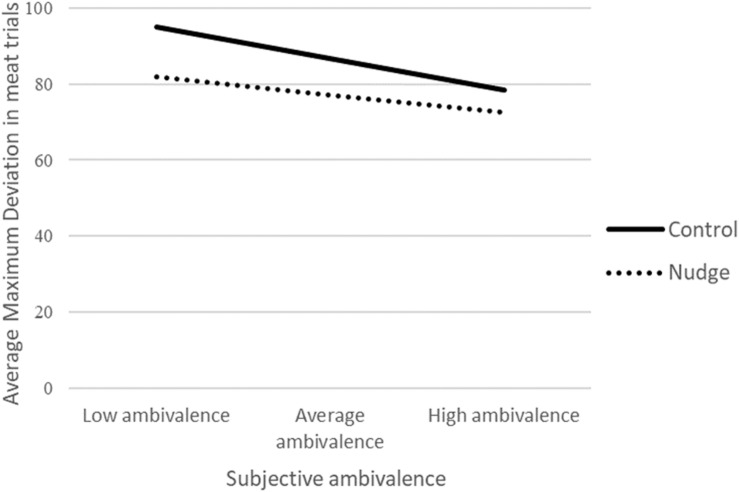
Results Study 2 on uncertainty.

To test our second hypothesis that a social proof nudge facilitates decision-making a repeated measures ANOVA was performed comparing the maximum deviation for the meat trials between the control and the nudge condition. Self-reported ambivalence was added as a covariate. As hypothesized, there was a main effect of condition, participants showed less uncertainty in the nudge condition (*M* = 77.13, *SD* = 35.54) than in the control condition, *M* = 86.43, *SD* = 40.61, *F*(1,95) = 8.53, *p* = 0.004, $\eta_{\text{p}}^{2}$ = 0.08. These findings are in support of the idea that a nudge can facilitate a decision. There was a marginally significant main effect for self-reported ambivalence, *F*(1,95) = 3.28, *p* = 0.073, $\eta_{\text{p}}^{2}$ = 0.03. The Condition × Self-reported ambivalence interaction effect was not significant for maximum deviation, *p* = 0.165, $\eta_{\text{p}}^{2}$ = 0.02, indicating that the nudge facilitated the decision for both highly and less self-reported conflicted individuals. Figure 6 shows the average maximum deviation in the meat trials across conditions for −1 *SD*, the average and +1 *SD* of subjective ambivalence.

For exploratory purposes we also examined the effect of the nudge on response time. A repeated measures ANOVA was performed comparing the response times for the meat trials between the control and nudge condition. Self-reported ambivalence was again added as a covariate. There was a main effect of condition, participants were faster to respond in the nudge condition (*M* = 1379.33, *SD* = 429.17) compared to the control condition, *M* = 1541.14, *SD* = 439.65, *F*(1,95) = 19.87, *p* < 0.001, $\eta_{\text{p}}^{2}$ = 0.17. There was no significant main effect of self-reported ambivalence, *p* = 0.466. The Condition × Self-reported ambivalence interaction was marginally significant, *F*(1,95) = 19.78, *p* = 0.061, $\eta_{\text{p}}^{2}$ = 0.04. This indicates that response time was affected by the nudge, but that this did not vary due to feeling conflicted about eating meat.

### Discussion

The second study showed that when participants feel more conflicted about eating meat (i.e., those who had less clear prior preferences) chose less meat products in the presence of a social proof nudge compared to the situation in which there was no nudge. These results provided support for the first hypothesis that a nudge influences people’s decisions in the absence of strong prior preferences. The results also show support for the second hypothesis that a nudge facilitates the choice. The reduction in uncertainty by the nudge was unrelated to participants’ self-reported conflicting preferences (i.e., self-reported ambivalence).

## General Discussion

Building on prior research showing that nudges are ineffective when decision makers have strong specific antecedent preferences, we tested the hypothesis that a nudge would be particularly effective in the *absence* of a clear preference. The absence of a clear preference was conceptualized as indifference in study 1, and as conflicting preferences in study 2. Moreover, we tested the hypothesis that a nudge would facilitate the choice by reducing people’s uncertainty, as implicitly assessed by measuring their mouse movements while they made their choices. In line with the first hypothesis, both studies demonstrated that the social proof nudge was effective in guiding people’s choices when they did not have a clear prior preference; steering color categorization toward green in study 1 and steering the decision concerning meat products toward “reject” in study 2 for the participants with conflicting preferences about consuming meat. The second hypothesis pertaining to the facilitation effect of the nudge was supported in study 2 but not in study 1. Together, the results from these studies suggest that a nudge is effective in guiding people’s choices particularly when they do not know what to choose and that a nudge has the potential to reduce uncertainty.

The effectiveness of a social proof nudge relies on people showing conformity behavior, that is, do what other people apparently did. The term ‘social proof’ stems from Cialdini (1984), who introduced it in the field of behavioral economics and marketing. The social proof nudge is derived from social influence theories that were particularly in vogue in the beginning of the previous century (e.g., Sherif, 1935; Asch, 1952). A typical social influence study consisted of a participant making judgments about properties of a particular stimulus (i.e., length, duration, movement, color, etc.), while a group of confederates would deliberately give the incorrect answer. When the participants would adapt their answer to that of the group, conformity behavior was said to occur. Deutsch and Gerard (1955) distinguished two powerful motives that lead people to conform; the desire to be right, (i.e., informational social influence), and the desire to be liked (i.e., normative social influence). A meta-analysis has shown that particularly conformity behavior as a result of normative social influence has changed over the decades; the more individualistic the culture is the less people are likely to show conformity behavior (Bond and Smith, 1996). However, in support of the informational account of social influence, Tversky and Kahneman (1974) labeled following the descriptive norm as one of the heuristics (i.e., rules of thumb) that people might fall back on in the case of uncertainty, i.e., when others have chosen a particular choice one can trust that this is proof of what is considered the ‘right choice.’

The informational social influence account can also best explain the results of the current studies. In study 1 the extent to which people were certain about their choice (i.e., had a clear preference) could be manipulated by using clear versus ambiguous colors. Color categorization relies primarily on perceptual and categorization processes (e.g., Huette and McMurray, 2010; Webster and Kay, 2012), however, the ambiguous colors simulated a typical decision making situation as participants had to choose between two conflicting options. One could argue that there is an objective “correct” choice for the categorization of the ambiguous colors (e.g., Kelly, 1943). This might have triggered the desire to be right in some participants, who as a result assessed all available information. In study 1 we unexpectedly found that the nudge was only effective in promoting choices for ‘green’ while the same nudge did not work to steer people toward choosing ‘blue’ when categorizing ambiguous colors. Close scrutiny of the results indicated that the critical stimuli might have been more optically green than blue. This could have invoked a slight preference for green, thereby inducing perhaps the phenomenon of *doubt* in the critical trials rather than indifference. Doubt is characterized by having a slight preference or inclination for one particular option but lacking the confidence to base a decision on it (Koriat et al., 1980). When viewing the results from study 1 as caused by doubt, i.e., a slight preference for green, it can explain why participants in the social proof green condition found the nudge helpful; it helped confirm their initial idea (Lamberton et al., 2013). These findings are in line with those of a recent study where information on peers was used to encourage employees to contribute to the 401k plan (Beshears et al., 2015). It was shown that social proof information lead to higher contributions for employees who were already in the plan with a low contribution, but had no effect for those who were not already enrolled. When the social proof information does not match the (slight) preference it might be regarded as irrelevant, (however, see Calluso et al., 2017). We expected that the inconsequential task of color categorization would be sufficient to invoke indifference, however, it could be argued that true indifference was demonstrated by the participants who failed to observe the social proof bar in the nudge conditions. The absence of the desire to be right could also explain why some participants in study 1 did not notice the social proof bar.

In study 2 the ‘correct’ choice depended on the perceived social reality of the participant, i.e., to what extent the choice for selecting a meat product would be approved by others. The effect of the nudge in study 2 was stronger for participants who experienced higher self-reported ambivalence to meat, i.e., these participants were more likely to conform to the implied descriptive norm to reject meat. This could be explained because their “social reality” might be different. Ambivalence is characterized by both strong preferences in favor and strong preferences against a particular choice, and this makes a decision important (Van Harreveld et al., 2009). So, for people who experience high ambivalence the decision might feel more as if there is a “correct” choice. Whereas for people who experience low ambivalence, the decision might not be perceived as having a “correct” response and therefore the desire to be right might not be there. This can explain why the effect of the social proof nudge was stronger for participants who experienced stronger conflicting preferences. In both studies the social proof nudge serves as a source of information when there is uncertainty about the correct answer.

In the current studies we operationalized facilitation by a nudge specifically as reduced uncertainty measured with the mouse-tracker. Even though we did not find support for the hypothesis that the nudge reduced uncertainty in study 1, we did observe that the participants who indicated to find the nudge helpful had considerably higher uncertainty scores on the critical trials compared to the participants who did not find the nudge helpful. In study 2 on the other hand, we observed an overall facilitation effect of the nudge (i.e., reduced uncertainty). This was not moderated by the magnitude of self-reported conflicting preferences. As most participants reported to feel at least somewhat conflicted about eating meat, it might be that the range of self-reported ambivalence was too small to find a difference between people who did and did not experience conflict, as we would theoretically predict. Based on the current studies we can carefully conclude that the extent to which people feel uncertainty in relation to a choice is a relevant factor to take into account when designing nudge interventions.

In the nudge literature there is currently no consensus about what facilitation exactly entails (e.g., Marchiori et al., 2017). In this article we operationalized facilitation of the decision as reduced uncertainty. However, note that even though uncertainty about the right choice option will make a decision difficult (i.e., resolving the uncertainty would make the decision easier), a person can be certain that a particular choice option is right and still find it difficult to actually make that decision (e.g., procrastination behavior). In these studies we relied on the mouse-tracker to assess whether a nudge made a decision easier, with the main advantage that it provides an insight in the uncertainty of the decision even when people are not consciously reflecting on this decision (e.g., Schneider et al., 2015). Next to maximum deviation, a variable that is typically used in ambivalence research (e.g., Schneider and Schwarz, 2017), we reported the findings on response time. Even though the findings on response time mostly corroborated those on maximum deviation, more research is necessary that further explores the use of mouse-tracker variables with different types of decisions. Considering that maximum deviation is a representation of the pull toward the alternative option, it may be most appropriate for decisions that indeed invoke conflict (i.e., where participants are drawn toward both options). Doubt, on the other hand, might be solely attributable to the characteristics of one choice option and independent of the alternative. To illustrate, when deciding on the means of transportation to an important event, the presented choice might be between not going at all, and the unappealing option of driving for 18 h. Even though the first option is not viable, you might be hesitant to accept the long drive. In the case of doubt maximum deviation may not accurately represent the choice uncertainty: there is little pull to the option of not going, nonetheless, the decision is made with little certainty.

While further research in the facilitation effect of nudges is warranted, mouse-tracker measurement is not feasible for all types of nudges since it requires a computer task setting. In the current studies we consider the facilitation effect as a side-effect of the nudge, but for many types of nudges it is assumed to be the working mechanism. For example, when nutrition information is presented using a traffic light system it becomes presumably easier to understand; or a staircase that is made more salient may become easier to find. Vigilance about these assumptions is warranted, however. To illustrate, despite the intention to simplify nutritional information, consumer studies have shown that traffic labels do not necessarily make it easier to judge the healthiness of products (Feunekes et al., 2008). Finding ways to critical test the assumptions related to nudging remains key to be able to optimize nudge interventions.

### Limitations and Future Studies

These studies serve as a proof of principle to measure how the absence of a strong preference affects the effectiveness of nudges. Future research needs to replicate these findings with improved external validity. Although it is unlikely that any choice architect thinks that people would be better off on the long-run if they would choose blue over green in a computer task, it is an operationalization of a situation in which the decision maker is only marginally interested in the outcome of the decision, which is the case, for example, in water conservation behavior (Onyenankeya et al., 2015). The current studies both used a social proof nudge, and since the preference framework is derived from a wide range of nudges types, future research should test the effectiveness also with other types of nudges, specifically in the absence of clear preferences. In study 1 a considerable number of participants did not notice the social proof nudge, a probable explanation is that “the need to be right” was not active in these participants (Deutsch and Gerard, 1955). Future studies should investigate whether indifferent individuals would be responsive to nudges that do not provide information, but instead rely on affordances, such as positioning nudges. Recent studies by Hütter and Fiedler (2019) demonstrated that even arbitrary anchors influenced decisions, however, in line with our findings, the degree of integration of actual advice vs. arbitrary anchors was related to participants’ subjective confidence. An important limitation related to study 2 was that order of the blocks was not randomized. The main reason for not counterbalancing the blocks was that the control condition might become “contaminated” by the carry-over effect of the implied social norm to reject meat in the nudge condition. The current studies also give rise to new research questions. While we tested the lack of a clear preference specified as indifference and conflicting preferences, conceptually it would be possible to distinguish a third type: doubt. It should be empirically tested how nudges affect doubt, where the interesting possibility arises that a nudge may lead to *more* rather than less doubt when it would go against people’s (slight) initial preference.

## Conclusion

Increasing our understanding of how people’s antecedent preferences influence the effectiveness of nudges allows for better nudge interventions that are more specifically directed toward certain target groups and/or certain types of choices. One implication in this regard would be that certain behavior might be more ‘nudgeable’ in certain populations or settings. For example, while repositioning healthy snacks might work to stimulate healthy choices at the train station, because people want to eat *something* to curb their hunger, a similar intervention might not be effective in a movie theater if people would have stronger preferences to treat themselves to something unhealthy. These studies are contributing to the literature not only because this is the first time that empirical studies have shown that in the absence of a clear preference a nudge is effective in steering the decision, but also because they are one of the first to directly test the facilitation effect of nudges.

While nudging has shown to be effective in influencing a decision, the current work implicates an important role for other interventions such as education, which shape preferences in the first place. The combination may be key to sustainable behavioral change (Mols et al., 2015).

## Data Availability Statement

The datasets generated for this study are available on request to the corresponding author.

## Ethics Statement

The studies involving human participants were reviewed and approved by Facultaire Ethische Toetsingscommissie (FETC), Social and Behavioural Sciences, Utrecht University. The patients/participants provided their written informed consent to participate in this study.

## Author Contributions

TV, DR, and FK conceptualized the research ideas. TV and JB developed the research designs. TV collected the data. TV analyzed and interpreted the data in consultation with FK, JB, and DR. TV drafted the manuscript. FK, JB, and DR provided critical feedback on the manuscript. All authors contributed to the manuscript and approved the submitted version.

## Conflict of Interest

The authors declare that the research was conducted in the absence of any commercial or financial relationships that could be construed as a potential conflict of interest.

## Footnotes

1 The exact stimuli and summary of the pilot study are available in the Supplementary Files.

2 Ten extreme outliers needed to be removed, outliers were defined as more than 1.5 the InterQuartileRange based on Tukey’s Hinges percentiles.

3 Due to a technical error the data was lost for three trials of meat products in the social proof condition, therefore the dependent variable is constructed by the percentage of selected products in the critical trials instead of absolute numbers.
