# Supplementary material for: When in Doubt, Follow the Crowd? Responsiveness to Social Proof Nudges in the Absence of Clear Preferences
Source: Front Psychol. 2020 Jun 18;11:1385. doi: 10.3389/fpsyg.2020.01385 (PMC7325907; doi:10.3389/fpsyg.2020.01385)
Supplement: Supplementary file 1 [file Data_Sheet_1.PDF]

Supplementary file Pilot ambiguous colours

Pilot study

Method

Participants. A convenience sample of 62 participants (88.7% female) were recruited on social media to determine which colours were suitable as critical stimuli. The average age was 24.69 (*SD* = 7.68). Participation was completely voluntary and participants received no compensation for their time.

Procedure. Participants completed the online study in Qualtrics. After providing informed consent, 33 colours from the Farnswerth-Musell 100-hue test were presented in a random order (see Figure 1). For each stimulus two measures of uncertainty were assessed: objective ambivalence and subjective uncertainty. Finally, participants indicated their gender and age and were thanked for their participation.

Objective ambivalence. Participants indicated for each colour how blue and how green they found this colour on a visual analogue scale ranging from 0% to 100%. Objective ambivalence was calculated with the formula by Thompson, Zanna and Griffin (1995) by adapting positive and negative to blue and green $[(B + G)/2] - |B - G|$. A higher score indicates stronger ambivalence.

Subjective uncertainty. Subsequently, participants were asked to decide whether this colour was blue or green. Lastly they were asked to indicate how certain they were of this decision on a visual analogue scale ranging from 0 (not at all) to 100 (absolutely certain). A lower score indicates more uncertainty.

Results

The results are depicted in Figure 2 and 3. No formal analyses were performed.

Figure 1. Selected stimuli

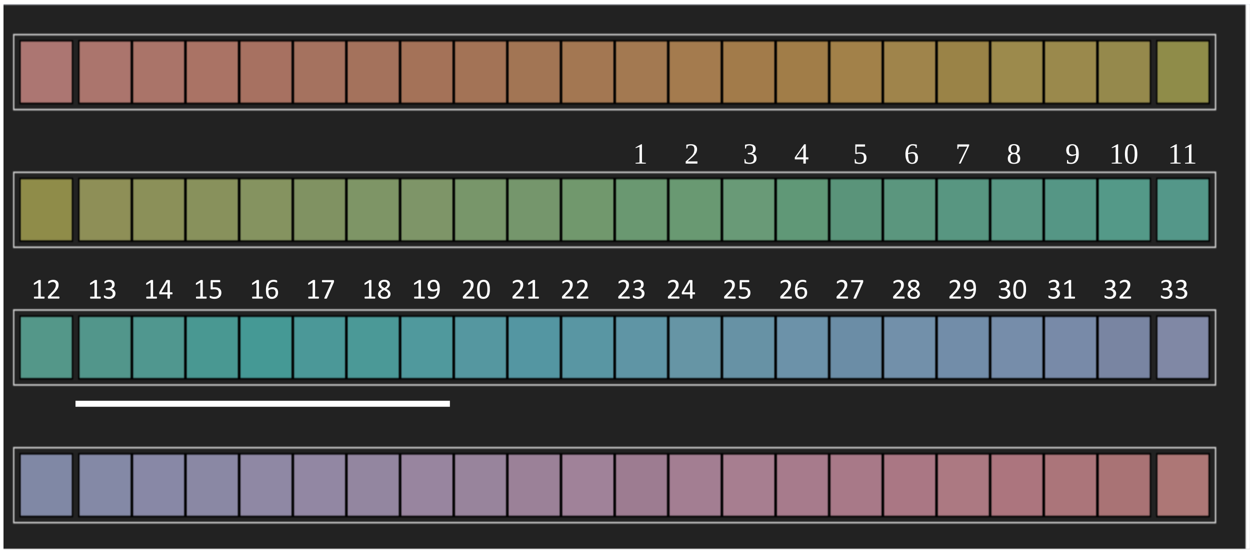

Note. The underlined stimuli were selected as critical stimuli for Study 1 from the range of numbered stimuli.

Figure 2. Results of Objective ambivalence pilot study

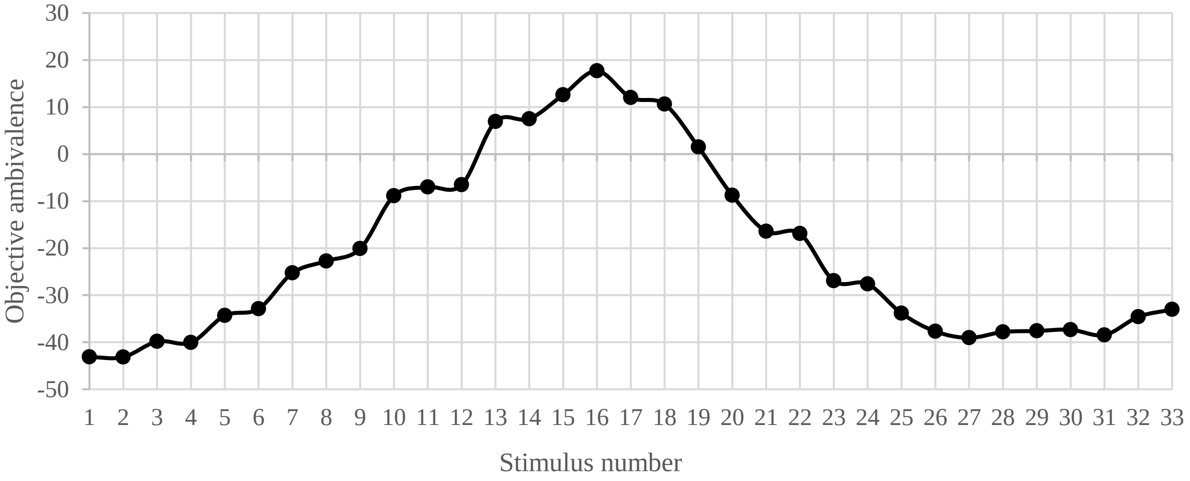

Figure 3. Result self-report certainty pilot study

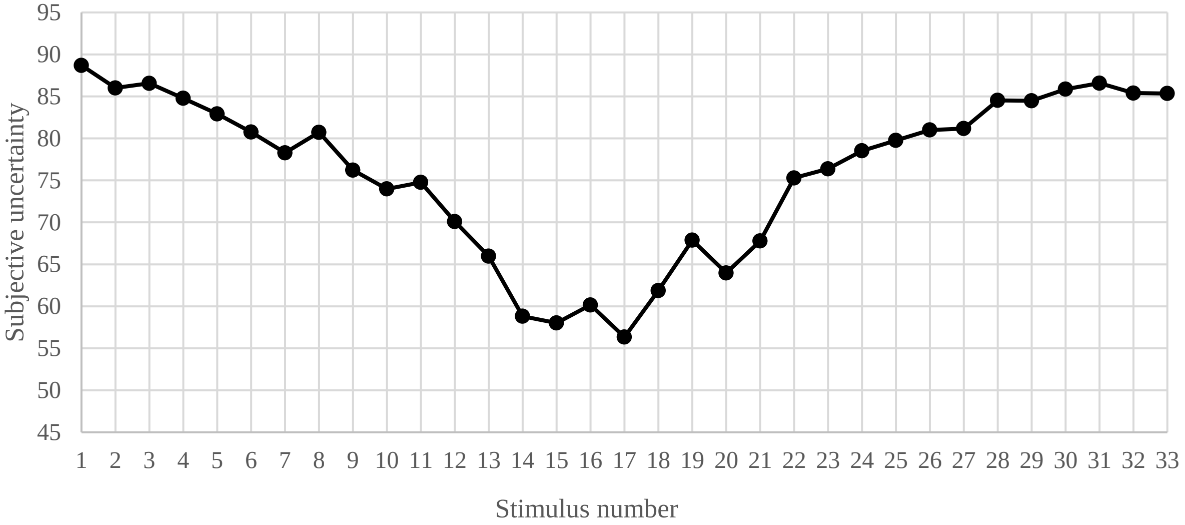

References

Thompson, M. M., Zanna, M. P., & Griffin, D. W. (1995). Let's not be indifferent about (attitudinal) ambivalence. *Attitude strength: Antecedents and consequences*, *4*, 361-386.
